# Exploration of the Association between Dietary Fiber Intake and Hypertension among U.S. Adults Using 2017 American College of Cardiology/American Heart Association Blood Pressure Guidelines: NHANES 2007–2014

**DOI:** 10.3390/nu10081091

**Published:** 2018-08-15

**Authors:** Baoqi Sun, Xiaoyan Shi, Tong Wang, Dongfeng Zhang

**Affiliations:** 1 Department of Epidemiology and Health Statistics, School of Public Health, Qingdao University, No. 38 Dengzhou Road, Qingdao 266021, China; SunBaoQi1214@163.com (B.S.); wangtong0106@126.com (T.W.); 2 Qingdao Center for Disease Control and Prevention, No. 17 Shandong Road, Qingdao 266033, China; shixiao0772@sina.com

**Keywords:** hypertension, high blood pressure, diet, dietary fiber, dose-response

## Abstract

This study aimed to explore the association between dietary fiber intake and hypertension risk using 2017 American College of Cardiology/American Heart Association Blood Pressure Guidelines. Data from the National Health and Nutrition Examination Survey 2007–2014 were used in this study. Dietary fiber data were obtained through two 24-h dietary recall interviews. Hypertension was defined as systolic blood pressure (SBP) ≥ 130 mmHg or diastolic blood pressure (DBP) ≥ 80 mmHg or treatment with hypertensive medications. Logistic regression models and restricted cubic spline models were applied to evaluate the associations between dietary intakes of total, cereal, vegetable, and fruit fiber and hypertension. A total of 18,433 participants aged 18 years or older were included in the analyses. After adjustment for age, gender, body mass index (BMI), race, educational level, smoking status, family income, and total daily energy intake, compared with the lowest tertile, the odds ratios (95% confidence intervals) of hypertension for the highest tertile intakes of total, cereal, vegetable, and fruit fiber were 0.62 (0.52–0.75), 0.80 (0.67–0.96), 0.82 (0.69–0.98), and 0.86 (0.71–1.04), respectively. Dose-response analyses revealed that the risk of hypertension was associated with total fiber intake in a nonlinear trend, while the relationships were linear for cereal and vegetable fiber intakes. Our results suggested that the intakes of total, cereal, and vegetable fiber, but not fruit fiber, were associated with a decreased risk of hypertension in U.S. adults.

## 1. Introduction

Hypertension, defined by the American College of Cardiology (ACC) and American Heart Association (AHA) as blood pressure above 130/80 mmHg [1] in 2017, is a public health issue. The number of people with hypertension increased from 600 million in 1980 to 1 billion in 2008 and is expected to reach 1.5 billion by 2025, accounting for almost one-third of the world’s population [2]. The prevalence of hypertension is 46% in African adults aged 25 and above [2], and approximately 31% of U.S. adults aged ≥18 years have hypertension [3]. Complications of hypertension cause 9.4 million deaths each year worldwide, and at least 45% of deaths due to heart disease and 51% of deaths due to stroke are attributed to hypertension. Hypertension is considered the major risk factor for the global disease burden [4]. Thus, it is indispensable to pay attention to the prevention and control of hypertension.

Epidemiologic evidence has revealed the associations between hypertension and dietary factors, such as fruit, vegetable, oats, and buckwheat [5,6,7,8]. As one beneficial dietary nutrient mainly from cereals, vegetables, and fruits [9], dietary fiber has been reported to have protective effects on several diseases, such as cardiovascular disease and stroke [10,11,12,13]. Meanwhile, the relationship between dietary fiber and hypertension has also been reported [6,14,15,16,17,18,19,20,21,22,23]. Some studies have reported that high dietary fiber consumption decreased the risk of hypertension or blood pressure (BP) [6,14,15,16,17,18,21], although other research studies reported that fiber intake was not significantly associated with hypertension [19,20]. Therefore, the results of studies on the association between dietary fiber and hypertension are not completely consistent. To our knowledge, since the 2017 High Blood Pressure Clinical Practice Guideline was released [1], there has not been studies investigating the association between dietary fiber and hypertension. Additionally, most existing epidemiological research studies on the relationship between dietary fibers and hypertension just focused on the total fiber, and only a limited number of studies explored the relationship between hypertension and dietary fiber from cereal, fruit, and vegetable in the same study [17]. Furthermore, few studies have investigated the dose-response relationship between dietary fiber intake and hypertension. Therefore, using the data from the National Health and Nutrition Examination Survey (NHANES) 2007–2014, and the new 2017 ACC/AHA hypertension guidelines [1], we evaluated the associations and dose-response relationship between intakes of total, cereal, vegetable, and fruit dietary fiber and hypertension in U.S. adults.

## 2. Materials and Methods

### 2.1. Study Population

**Figure 1:**
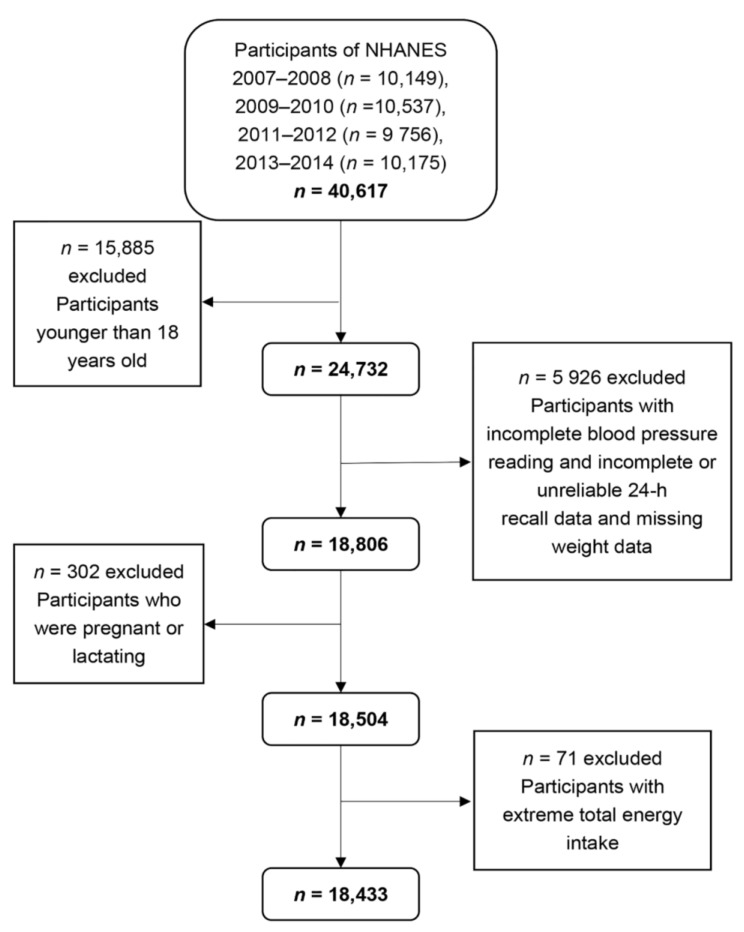
Flow chart of the screening process for the selection of eligible participants. NHANES, National Health and Nutrition Examination Survey.

NHANES aims to assess the health and nutritional status of the U.S. population and adopts a complex multi-stage probabilistic sampling design to select representative samples of the civilian non-institutional U.S. population. NHANES participants were first interviewed in their homes and then completed the health examination in a mobile examination center (MEC) [24]. The NHANES database is a publicly available dataset for use by researchers around the world; data are released in 2-year cycles and can be downloaded from the NHANES website [25]. A total of 40,617 individuals participated in NHANES during 2007–2014. We selected 24,732 individuals who were 18 years of age or older. Among them, we excluded participants with incomplete BP readings (*n* = 1888), incomplete or unreliable 24-h recall data (*n* = 3874), and missing weight data (*n* = 164). We also excluded pregnant (*n* = 194) or lactating (*n* = 108) females. Moreover, individuals were omitted who had extreme total energy intakes of <500 or >5000 kcal/day for females, and <500 or >8000 kcal/day for males (*n* = 71). Ultimately, this study contained a total of 18,433 participants aged 18 years or older (9015 men and 9418 women) (Figure 1). As NHANES is a publicly available dataset, the present study was exempt from approval by an institutional review board. All participants provided informed consents both before the interview and examination stages.

### 2.2. Blood Pressure Measurements

According to the American Heart Association and the NHANES procedures [1,26], all blood BP determinations (systolic and diastolic) were measured in the MEC. After sitting quietly for five minutes, certified examiners measured each participant’s seated blood pressure three times using a mercury sphygmomanometer. A fourth attempt was made if a BP measurement was interrupted or incomplete. We calculated means of systolic blood pressure (SBP) and diastolic blood pressure (DBP).

### 2.3. Definition of Hypertension

First, participants with self-reported use of BP-lowering medication to reduce BP were considered as hypertensive irrespective of the BP value [27]. Second, following the 2017 high blood pressure clinical practice guideline, other participants were divided into non-hypertension group (SBP < 130 mmHg and DBP < 80 mmHg, and hypertension group (SBP ≥ 130 mmHg and/or DBP ≥ 80 mmHg) [1].

### 2.4. Dietary Fiber Intake

Dietary fiber intake was assessed by two 24-h dietary recall interviews conducted by trained dietitians. The first dietary interview was collected in the MEC and the second interview was collected by telephone 3 to 10 days later. Nutrient intakes were calculated according to the U.S. Department of Agriculture’s Dietary Research Food and Nutrition Database for Dietary Studies [28]. Dietary fiber from different types of food was determined according to the food code. In this study, the dietary fiber intakes from both dietary recall interviews were averaged and adjusted to the body weight. Dietary fiber intakes (g/kg/day) were divided into tertiles. It should be noted that the total dietary fiber included fiber from supplements.

### 2.5. Other Potential Factors for Hypertension

In addition to dietary fiber intake, we investigated the influence of potential confounding factors., which included: age (18–39 years, 40–59 years, and ≥60 years), gender (male and female), race (Mexican American, other Hispanic, non-Hispanic White, non-Hispanic Black, and other race), educational level (below high school, high school, and above high school), annual household income (<$20,000, $20,000 to <$50,000, $50,000 to <$75,000, and ≥$75,000), body mass index (BMI) (normal: <25 kg/m^2^; overweight: 25 to <30 kg/m^2^; obese: ≥30 kg/m^2^), total energy intake and smoking status (never, never smoked or smoked <100 cigarettes in life; current, smoked ≥100 cigarettes in life and currently smoking; former, smoked ≥100 cigarettes in life and currently no longer smoking). Total energy intake was calculated by aggregating daily energy intake from diet and dietary supplements.

### 2.6. Statistical Analysis

Student’s *t*-tests were used to compare the mean values between participants with and without hypertension. Chi-square tests were used to compare the percentages of categorical variables between individuals with and without hypertension. Binary logistic regression models were used to analyze the association between hypertension and intakes of total, cereal, vegetable, and fruit fiber. In multivariate logistic regressions, model 1 adjusted for age and sex, and model 2 further adjusted for race, educational level, annual household income, BMI, total energy intake, and smoking status. Furthermore, stratified analyses were performed based on age (18 to <45 years, 45 to <65 years, and ≥65 years) and gender (male and female) to evaluate the relationship between total fiber intake and hypertension. The lowest tertile of dietary fiber intake was used as the reference group. The odds ratios (ORs) and 95% confidence intervals (CIs) were calculated from logistic regression analyses. After one percent abnormal values before and after were rejected, the dose-response relationship was assessed by binary logistic regression model with the use of restricted cubic spline function with three knots located at the 5th, 50th, and 95th percentiles of the exposure distribution. To increase the authenticity of the observed association, the restricted cubic spline model adjusted for the same confounding factors as those adjusted in the logistic regression model 2. The *p*-value for non-linearity was calculated by testing the value of the coefficient of the second spline of zero. All statistical analyses were conducted using Stata 15.0. To conduct a nationally representative estimate, appropriate sampling weights, primary sampling unit, and strata information were considered in this study. All reported probabilities (*p*-values) were two-sided with *p* < 0.05 considered as significant.

## 3. Results

Table 1 summarizes the information of the sample that included a total of 18,433 eligible participants. The prevalence of hypertension was 44.93%. The percentage of participants with hypertension was higher in the over 60 years group. Hypertension was more likely to occur in males and Non-Hispanic Black participants. Compared with non-hypertensive participants, those with hypertension received a lower level of education and income. Those with hypertension were more likely to smoke and be obese. Moreover, those without hypertension consistently consumed more dietary fiber and dietary fiber subtypes than those with hypertension (Table 1).

The weighted ORs (95% CIs) of hypertension according to tertiles of total, cereal, vegetable, and fruit fiber for all participants are shown in Table 2. In univariate logistic regression analyses, total and cereal fiber were associated with a decreased risk of hypertension. Compared with the lowest tertile, the ORs of hypertension for the highest tertile intake of vegetable and fruit fiber were 0.86 (0.76–0.97) and 0.82 (0.71–0.95), respectively. After adjustment for age and sex (model 1), the ORs of hypertension indicated that all levels of total, cereal, vegetable, and fruit fiber were associated with a decreased risk of hypertension. After further adjustment for total energy intake, educational level, race, annual household income, smoking status, and body mass index (model 2), total, cereal, and vegetable fiber intakes were inversely associated with the risk of hypertension. However, the protective effect of fruit fiber intake on hypertension was no longer significant after adjustment for more covariates.

The association between total fiber intake and hypertension in stratified analyses is displayed in Table 3. In stratified analyses by age, all levels of total fiber intake were associated with a decreased risk of hypertension in three models for participants aged less than 45 years. For participants aged 45 to 65 years, all levels of total fiber intakes were associated with a decreased risk of hypertension in the unadjusted model and model 1. In multivariate-adjusted model 2, the inverse association between the highest level of total fiber intake and hypertension was significant. For participants aged 65+ years, the inverse association with the risk of hypertension was significant in the highest tertile of total fiber intake in the unadjusted model and model 1, with ORs (95% CIs) of 0.64 (0.47–0.88) and 0.61 (0.45–0.81), respectively. However, the inverse association between total fiber intake and the risk of hypertension was not significant in multivariate adjusted model 2. In stratified analyses by sex, the inverse associations of total fiber intake with hypertension were similar in males and females and significant in the unadjusted model and the age-adjusted model; the ORs (95% CIs) of hypertension in the highest tertile of total fiber were 0.66 (0.52–0.84) and 0.58 (0.45–0.75), respectively, in model 2.

**Figure 2:**
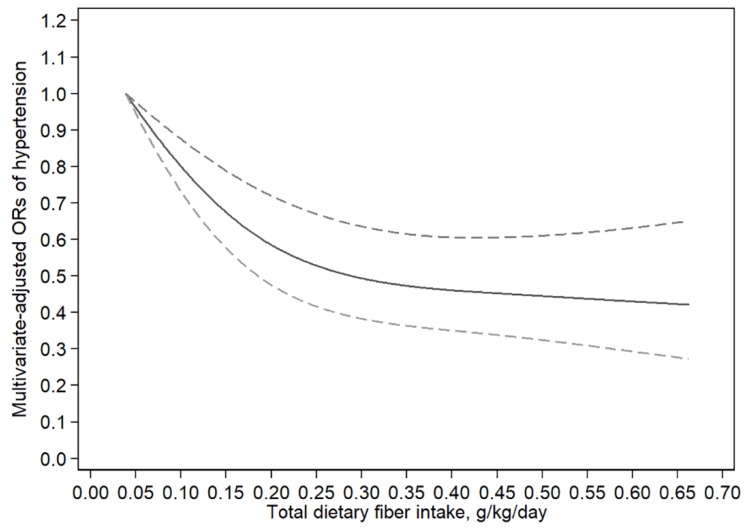
Examination of the dose-response relationship between total dietary fiber intake and the risk of hypertension by restricted cubic splines model. The lowest level of total fiber intake (0.071 g/kg/day) was used as the reference group. The restricted cubic splines model adjusted for age, gender, total energy intake, race, body mass index (BMI), annual household income, smoking status, and educational level. The solid line and dashed line represent the estimated ORs and the corresponding 95% confidence intervals, respectively. OR, odds ratio.

The result of the dose-response relationship analysis between total dietary fiber and hypertension is shown in Figure 2. A nonlinear negative correlation between total dietary fiber intake and hypertension was found (*P*_for nonlinearity_ < 0.01). With an increase of total fiber intake, there are no further reductions in hypertension risk beyond 0.35 g/kg/day (OR: 0.47; 95% CI: 0.36–0.62).

**Figure 3:**
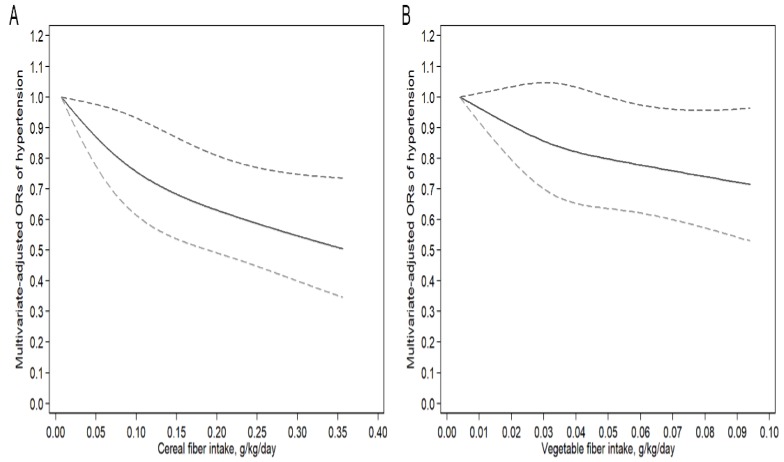
Examination of the dose-response relationship between dietary fiber intake and risk of hypertension by restricted cubic splines model. (**A**) Cereal fiber, *P*_for nonlinearity_ = 0.34; the lowest level of cereal fiber intake (0.02 g/kg/day) was used as the reference group. (**B**) Vegetable fiber, *P*_for nonlinearity_ = 0.12; the lowest level of vegetable fiber intake (0.004 g/kg/day) was used as the reference group. The restricted cubic splines model adjusted for age, gender, total energy intake, race, body mass index (BMI), annual household income, smoking status, and educational level. The solid line and dashed line represent the estimated ORs and the corresponding 95% confidence intervals. OR, odds ratio.

The dose-response relationships between cereal and vegetable fiber intakes and hypertension are presented in Figure 3. In restricted cubic spline models, cereal and vegetable fiber intakes were linear inversely associated with the risk of hypertension (*P*_for nonlinearity_ = 0.34 and 0.12, respectively). When the intakes of cereal fiber reach 0.016 g/kg/day (OR: 0.97; 95% CI: 0.94–0.99) and vegetable fiber reach 0.052 g/kg/day (OR: 0.76; 95% CI: 0.63–0.99), both dietary fibers show meaningful protective effects on the hypertension. The does-response relationship of fruit fiber was not carried out because no significant association was observed between dietary intake and BP in logistic regression model 2.

## 4. Discussion

In this cross-sectional study based on a nationally representative large-scale database and updated classification system for hypertension in 2017 [1], we found that the intakes of total, cereal, and vegetable dietary fiber, but not fruit fiber, were associated with a lower risk of hypertension in the US population aged 18 years and older. In the stratified analysis of age and sex, the total fiber intake was inversely related to hypertension in the 18 to <45 years old group, the 45–64 years old group, and in males and females. After adjustment for age, gender, total energy intake, educational level, race, annual household income, smoking status, and body mass index, the association still had statistical significance.

We also found that a nonlinear relationship was apparent for total fiber intake and hypertension. Higher intake of total fiber up to 0.35 g/kg/day was the threshold at which the line started to plateau. The risk of hypertension was reduced by 53% when the total fiber intake increased from 0.07 g/kg/day to 0.35 g/kg/day (Figure 2). Moreover, cereal and vegetable fiber intakes had linear inverse associations with the risk of hypertension.

Although we used new standards for hypertension [1], our findings of the protective effect of total, cereal, and vegetable dietary fiber on hypertension were consistent with previous studies from different populations [6,14,15,16,17,18,22,29]. Previous cross-sectional studies demonstrated that higher intakes of dietary fiber were associated with lower risk of hypertension [6,14,17,22]. A Mediterranean cohort study also revealed that cereal dietary fiber was inversely associated with a lower risk of hypertension [15]. Meta-analysis studies showed that increasing fiber intake in the general population may contribute to the prevention of hypertension [16,18].

We did not find a significant association of dietary fiber from fruits on hypertension. However, fruit fiber has been largely recognized as a protective factor in hypertension [29,30]. A cross-sectional study showed the lowering effect of fruit fiber intake on blood pressure level based on a sample of 805 men aged 40–69 years who were free from clinical hypertension [30]. Additionally, a prospective study demonstrated that fiber from fruit had a significant inverse association with the risk of hypertension among 30,681 white U.S. males aged 40–75 years [29]. The inconsistent results between our study and the abovementioned studies may be explained by differences in demographic characteristics and methodology. First, both studies were conducted in males aged 40 years old and above. Second, for the above studies, the frequency of BP measurements was twice [30] or based on self-reported BP data [29]. Third, fiber intakes were calculated by semiquantitative food-frequency questionnaire in two studies, whereas our findings were derived from three averaged BP measurements and two more detailed 24-h dietary recall interviews conducted by trained interviewers. In addition, the participants in our study were males and females over 18 years old, so further exploration of the relationship between fruit fiber and hypertension based on age and gender stratification analysis is needed.

Our study has several advantages. First, since the update of the standard for hypertension in 2017, we conducted the first study to explore the relationship between total, cereal, vegetable, and fruit dietary fiber and the risk of hypertension, using the new standard for hypertension. Second, we investigated the dose-response relationship between fiber intake and the risk of hypertension.

However, our study also has some limitations. First, we cannot infer causal interpretations of the relationship between dietary fiber intake and hypertension risks because of the cross-sectional design of the study. Second, although a number of potential confounding factors were controlled, we cannot exclude the possibility of residual confusion caused by unmeasured confounding factors. Third, our sample population contains many people who were previously diagnosed with hypertension or were currently taking antihypertensive medications. The dietary patterns of these individuals may have changed, which may have an impact on the outcome. Fourth, we were unable to estimate separate associations between hypertension risks and soluble or insoluble fiber, which may have differential impacts on hypertension risks. Moreover, the two 24-h diet recalls used in the study may be affected by recall bias and cannot accurately reflect individuals’ usual intake. Moreover, we cannot infer the mechanisms behind the reverse association between dietary fiber consumption and the risk of hypertension as an epidemiological study.

## 5. Conclusions

In conclusion, the intakes of total, cereal, and vegetable fiber, but not fruit fiber, were associated with lower risk of hypertension in U.S. adults in this cross-sectional study. The risk of hypertension gradually decreased as total dietary fiber intake increased until up to 0.35 g/kg/day. Therefore, it might be advantageous to select fiber-rich foods to prevent and control hypertension.

## Figures and Tables

**Table 1:** Characteristics of participants by hypertension, NHANES 2007–2014, adults ≥18 years of age.

	Non-Hypertension	Hypertension	*p* Value
**Number of Participants (%) ^1^**	9458 (55.07)	8975 (44.93)	
**Age group (%) ^1^**			<0.01
18–39 years	5212 (80.79)	1244 (19.21)	
40–59 years	2956 (51.50)	3026 (48.50)	
≥60 years	1290 (24.31)	4705 (75.69)	
**Sex (%) ^1^**			<0.01
Male	4403 (52.89)	4612 (47.11)	
Female	5055 (57.64)	4363 (42.36)	
**Race (%) ^1^**			<0.01
Mexican American	1670 (68.25)	1041 (31.75)	
Other Hispanic	1083 (66.74)	767 (33.26)	
Non-Hispanic White	4108 (53.47)	4253 (46.53)	
Non-Hispanic Black	1584 (45.59)	2302 (54.41)	
Other race	1013 (62.27)	612 (37.73)	
**Educational level (%) ^1^**			<0.01
Below high school	2197 (51.06)	2434 (48.94)	
High school	2040 (51.15)	2231 (48.85)	
Above high school	5213 (57.68)	4301 (42.32)	
**Household income (%) ^1^**			<0.01
<$20,000	1770 (50.81)	2066 (49.19)	
$20,000 to <50,000	3035 (52.04)	3033 (47.96)	
$50,000 to <75,000	1696 (54.77)	1608 (45.23)	
≥$75,000	2562 (59.38)	1929 (40.62)	
**Body mass index (%) ^1^**			<0.01
<25 kg/m^2^	3660 (70.65)	1850 (29.35)	
25 to <30 kg/m^2^	3113 (55.21)	2917 (44.79)	
≥30 kg/m^2^	2677 (41.57)	4178 (58.43)	
**Smoking Status (%) ^1^**			<0.01
Never	1981 (60.18)	1578 (39.82)	
Currently	5194 (57.02)	4623 (42.98)	
Former	1682 (43.11)	2708 (56.89)	
**Body weight (kg) ^2^**	78.04 (0.36)	87.37 (0.36)	<0.01
**Total energy intake (kcal/day) ^2^**	2146.50 (13.10)	2048.12 (13.42)	<0.01
**Total fiber intake (mg/kg/day) ^2^**	236.70 (2.94)	205.88 (2.51)	<0.01
**Cereal fiber intake (mg/kg/day) ^2^**	107.74 (1.56)	88.79 (1.32)	<0.01
**Vegetable fiber intake (mg/kg/day) ^2^**	48.91 (0.84)	45.18 (0.69)	<0.01
**Fruit fiber intake (mg/kg/day) ^2^**	43.52 (0.94)	38.80 (0.85)	<0.01

^1^ Number of participants and weighted percentage. Chi-square test was used to compare the percentage between participants with and without hypertension. ^2^ Weighted mean value and standard error (SE). Student’s *t*-test was used to compare the mean values between participants with and without hypertension.

**Table 2:** Weighted ORs and 95% CIs for hypertension according to tertiles of dietary fiber intake, NHANES 2007–2014, adults aged ≥18 years.

	Cases/Participants ^1^	Weighted Prevalence (%) ^2^	Crude ^3^	Model 1 ^3^	Model 2 ^3^
			OR (95% CI)	OR (95% CI)	OR (95% CI)
**Total fiber (g/kg/day)**					
<0.147	3343/6144	50.94	1.00 (Ref.)	1.00 (Ref.)	1.00 (Ref.)
0.147 to <0.245	3092/6144	46.15	0.83 (0.73–0.94) **	0.67 (0.58–0.78) **	0.82 (0.69–0.96) *
≥0.245	2540/6145	37.4	0.58 (0.51–0.64) **	0.42 (0.37–0.48) **	0.62 (0.52–0.75) **
**Cereal fiber (g/kg/day)**					
<0.057	3392/6108	50.65	1.00 (Ref.)	1.00 (Ref.)	1.00 (Ref.)
0.057 to <0.108	3032/6109	46.62	0.85 (0.76–0.95) **	0.76 (0.67–0.86) **	0.90 (0.78–1.03)
≥0.108	2503/6110	37.22	0.58 (0.52–0.65) **	0.55 (0.47–0.64) **	0.80 (0.67–0.96) *
**Vegetable fiber (g/kg/day)**					
<0.023	2877/5785	46.39	1.00 (Ref.)	1.00 (Ref.)	1.00 (Ref.)
0.023 to <0.050	2929/5787	46.09	0.99 (0.87–1.12)	0.84 (0.73–0.98) *	0.91 (0.78–1.07)
≥0.050	2694/5787	42.74	0.86 (0.76–0.97) *	0.65 (0.56–0.75) **	0.82 (0.69–0.98) *
**Fruit fiber (g/kg/day)**					
<0.018	2320/4563	47.12	1.00 (Ref.)	1.00 (Ref.)	1.00 (Ref.)
0.018 to <0.046	2328/4563	46.95	0.99 (0.88–1.12)	0.82 (0.71–0.95) *	0.94 (0.80–1.09)
≥0.046	2168/4565	42.27	0.82 (0.71–0.95) **	0.60 (0.50–0.72) **	0.86 (0.71–1.04)

CI, confidence interval; OR, odds ratio. ^1^ Hypertensive cases/number of participants in tertiles. ^2^ Hypertensive weighted prevalence (%) in tertiles. ^3^ Calculated using binary logistic regression, model 1 adjusted for age and gender, model 2 adjusted for age and gender, total energy intake, race, body mass index (BMI), annual household income, smoking status, and educational level. The lowest tertile of dietary fiber intake was used as the reference group. Results are survey-weighted. * *p* < 0.05; ** *p* < 0.01.

**Table 3:** Weighted ORs and 95% CIs for hypertension according to tertiles of total fiber intake, stratified by age and gender, NHANES 2007–2014, adults aged ≥18 years.

Total Fiber (g/kg/day)	Cases/Participants ^1^	Weighted Prevalence (%) ^2^	Crude ^3^	Model 1 ^3^	Model 2 ^3^
			OR (95% CI)	OR (95% CI)	OR (95% CI)
**18 ≤ age < 45 years**					
<0.147	882/2796	31.64	1.00 (Ref.)	1.00 (Ref.)	1.00 (Ref.)
0.147 to <0.245	550/2541	21.93	0.61 (0.51–0.73) **	0.55 (0.46–0.67) **	0.69 (0.55–0.88) **
≥0.245	398/2637	15.52	0.40 (0.33–0.48) **	0.33 (0.27–0.41) **	0.50 (0.38–0.67) **
**45 ≤ age < 65 years**					
<0.147	1397/2068	65.1	1.00 (Ref.)	1.00 (Ref.)	1.00 (Ref.)
0.147 to <0.245	1258/2055	58.67	0.76 (0.61–0.95) *	0.76 (0.60–0.93) *	0.88 (0.69–1.12)
≥0.245	1054/2093	47.36	0.48 (0.40–0.58) **	0.46 (0.38–0.56) **	0.69 (0.53–0.90) **
**Age ≥ 65 years**					
<0.147	1064/1280	81.39	1.00 (Ref.)	1.00 (Ref.)	1.00 (Ref.)
0.147 to <0.245	1284/1548	80.5	0.94 (0.66–1.35)	0.93 (0.66–1.30)	1.11 (0.75–1.66)
≥0.245	1088/1415	73.74	0.64 (0.47–0.88) **	0.61 (0.45–0.81) *	0.83 (0.53–1.29)
**Male**					
<0.147	1670/2883	54.19	1.00 (Ref.)	1.00 (Ref.)	1.00 (Ref.)
0.147 to <0.245	1560/2962	49.14	0.82 (0.69–0.96) *	0.72 (0.60–0.87) *	0.88 (0.71–1.10)
≥0.245	1382/3170	39.06	0.54 (0.46–0.64) **	0.45 (0.38–0.55) **	0.66 (0.52–0.84) **
**Female**					
<0.147	1673/3261	48.07	1.00 (Ref.)	1.00 (Ref.)	1.00 (Ref.)
0.147 to <0.245	1532/3182	43.32	0.83 (0.70–0.98) *	0.62 (0.52–0.75) **	0.74 (0.59–0.94) **
≥0.245	1158/2978	35.69	0.60 (0.51–0.70) **	0.38 (0.32–0.45) **	0.58 (0.45–0.75) **

CI, confidence interval; OR, odds ratio. ^1^ Hypertensive cases/number of participants in tertiles. ^2^ Hypertensive weighted prevalence (%) in tertiles. ^3^ Calculated using binary logistic regression, model 1 adjusted for age and gender, model 2 adjusted for age and gender, total energy intake, race, body mass index (BMI), annual household income, smoking status, and educational level. The corresponding stratified variables were excluded from the adjusted models. The lowest tertile of dietary fiber intake was used as the reference group. Results are survey-weighted. * *p* < 0.05; ** *p* < 0.01.
